# Clinical analysis of sarcopenia prevalence and its influencing factors in patients with Parkinson’s disease

**DOI:** 10.3389/fnagi.2025.1718723

**Published:** 2025-12-08

**Authors:** Remi Morimoto, Kazuo Kitagawa, Kenichi Todo, Mutsumi Iijima

**Affiliations:** 1 Department of Neurology, Tokyo Women’s Medical University School of Medicine, Tokyo, Japan; 2 Department of Neurology, Suita Municipal Hospital, Suita, Japan

**Keywords:** Parkinson’s disease, sarcopenia, gender, cognitive impairment, swallowing disturbance

## Abstract

**Background:**

Patients with Parkinson’s disease (PD) have a high risk of sarcopenia. Herein, we evaluated the prevalence of sarcopenia and factors associated with it in ambulatory patients with PD.

**Methods:**

Ambulatory patients with PD up to Hoehn and Yahr stage III were included and evaluated based on age, sex, disease duration, levodopa equivalent daily dose, cognitive impairment, swallowing disturbance, history of falling, the Japanese version of the movement disorder society-sponsored revision of the unified PD rating scale (MDS-UPDRS) parts I–IV, quality of life (QoL), and blood test (total protein, albumin, and anemia). Cognitive impairment was assessed using the Japanese version of Mini-Mental State Examination and the Japanese version of the Montreal Cognitive Assessment, whereas swallowing disturbance was assessed using the Japanese version of the Swallowing Disturbance Questionnaire. QoL was assessed using the Parkinson’s Disease Questionnaire (PDQ-8). Sarcopenia was diagnosed based on handgrip strength, five-time chair stand test, and skeletal muscle mass.

**Results:**

Overall, 97 patients with PD (55 males), with a mean age of 69.8 years and a mean disease duration of 7.3 years, were included. The prevalence of sarcopenia was 33.0%. There were significant differences between the sarcopenia and the non-sarcopenia groups in age, sex, swallowing disturbance, MDS-UPDRS part III total score, and the sub-items arising from a chair, postural stability, and global spontaneity of movement (*p* < 0.05). There was no association between the presence of sarcopenia and the PDQ-8 total and sub-item scores. The factors that contributed most to sarcopenia were being female, cognitive impairment, and swallowing disturbance.

**Conclusion:**

In clinical management, it is important to assess muscle strength and evaluate sarcopenia, particularly in patients with PD who have being female, cognitive impairment, or swallowing disturbance.

## 1 Introduction

Parkinson’s disease (PD) causes weight loss, fatigue, decreased activity, and slowed gait (Marinus et al., 2018), as well as sarcopenia, a loss of muscle mass that can lead to decreased muscle strength and physical function (Yazar et al., 2018; Peball et al., 2019). The general prevalence of sarcopenia is 7.5%–13.8% (Akune et al., 2014; Yoshida et al., 2014; Yoshimura et al., 2017), whereas that of sarcopenia in patients with PD is 17%–50%, depending on the subject and assessment method (Vetrano et al., 2018; Cai et al., 2021).

In 1989, Rosenberg posited the importance of age-related loss of skeletal muscle mass and promoted the term sarcopenia (Rosenberg, 1989). Sarcopenia, defined as the age-related loss of muscle mass and function, is increasingly recognized as a serious medical and economic problem in an aging society (Cruz-Jentoft et al., 2010) and has become a significant global public health focus (Shafiee et al., 2017). The causes of sarcopenia remain undetermined, and the prevailing theories consider it multifactorial, with the normal aging process increasing the risk of sarcopenia (Cruz-Jentoft et al., 2010).

Although previous reports have investigated the prevalence of sarcopenia in patients with PD (Ponsoni et al., 2023), the association between PD severity and sarcopenia (Vetrano et al., 2018), and changes in body composition and sarcopenia in patients with PD (Tan et al., 2018), there have been no reports of an association between sarcopenia and cognitive impairment, swallowing disturbance, or quality of life (QoL) in patients with PD. The Parkinson’s Disease Questionnaire-8 (PDQ-8), a shortened version of PDQ-39, was developed to reduce respondent burden and increase the convenience of use for patients with PD in clinical settings (Peto et al., 1998; Hagell et al., 2003; Chen et al., 2017).

PDQ-8 was constructed by taking one question from each domain of PDQ-39 (Peto et al., 1998; Chen et al., 2017). It is an established tool for assessing patients with PD that is frequently used in clinical studies (Martinez-Martin et al., 2011). Accordingly, in this study, we assessed QoL using PDQ-8 for convenience and examined the prevalence of sarcopenia in patients with PD and the factors affecting sarcopenia, including motor and non-motor symptoms and QoL in patients with PD, using the AWGS (2019 ASIAN working group for sarcopenia) diagnostic criteria.

## 2 Materials and methods

### 2.1 Participants

Ambulatory patients with PD up to Hoehn and Yahr (H-Y) stages I-III enrolled from December 1, 2021, to June 1, 2024, were included. As an inclusion criterion and for dementia screening, patients with a score ≤23 on the Japanese version of the Mini-Mental State Examination (MMSE-J) were excluded. PD was clinically diagnosed using the MDS clinical diagnostic criteria (Postuma et al., 2015).

### 2.2 Evaluation of clinical symptoms

Sarcopenia was diagnosed using the diagnostic criteria of AWGS (Chen et al., 2014). The AWGS diagnostic criteria are shown in Supplementary File 1.

In this study, physical performance was assessed using a five-time chair stand test, and appendicular skeletal muscle mass was assessed using a body component analyzer (InBody720^®^; InBody, Seoul, S. Korea). The InBody device is based on bioelectrical impedance analysis. Patients were evaluated based on age, sex, disease duration, H-Y stage, and history of falls. Cognitive impairment was assessed using the Japanese version of the Montreal Cognitive Assessment (MoCA-J), with scores of ≤25 on the MoCA-J considered abnormal. Swallowing disturbances were assessed using the Japanese version of the Swallowing Disturbance Questionnaire (SDQ-J) (Supplementary File 2): the SDQ-J total score of ≥11 points was classified as the swallowing disturbance group, and <11 points as the no swallowing disturbance group (Yamamoto et al., 2012).

The severity of PD was assessed using the H-Y stage, and clinical symptoms were evaluated using the Japanese version of the movement disorder society-sponsored revision of the unified Parkinson’s disease rating scale (MDS-UPDRS) parts I–IV (Kashihara et al., 2014). H-Y stage and MDS-UPDRS part III were assessed in the on-state. MDS-UPDRS part I focuses on non-motor symptoms in daily living, in which the patient or caregiver completes a 13-item questionnaire; part II focuses on motor symptoms in daily living, in which the patient or caregiver completes a 13-item questionnaire; part III covers objective motor symptoms, in which the assessor rates 18 motor symptoms; part IV covers motor complications, in which the assessor rates six questions regarding symptom variability and dyskinesia. The scores were evaluated as follows: 0, normal; 1, very mild; 2, mild; 3, moderate; 4, severe. QoL was assessed using the PDQ-8 (Peto et al., 1998; Supplementary File 3).

Each medication was evaluated using the levodopa equivalent daily dose (LEDD) (Jost et al., 2023). Biochemical factors (total protein [TP], albumin [Alb], and anemia) were evaluated using blood tests. An abnormal TP value was defined as ≤6.4 g/dl, and an abnormal Alb value was defined as ≤3.5 g/dl. The anemia group was defined as a group with a hemoglobin (Hb) value of <14 g/dL for male patients and <12 g/dL for female patients, and a hematocrit (Ht) value of <40% for male patients and <35% for female patients. All patients underwent magnetic resonance imaging to rule out other diseases. This study was approved by the Institutional Review Board of Tokyo Women’s Medical University (Approval No. 20210148). It was conducted in accordance with the Ethical Guidelines for Clinical Research in Japan and the Declaration of Helsinki. Written informed consent was obtained from all patients before the study commenced.

### 2.3 Statistical analysis

Data are expressed as the mean ± SD. JMP Pro statistical software (version 16; SAS Institute, Tokyo, Japan) was used for statistical analysis. The Wilcoxon test was used to compare age, disease duration, LEDD, MMSE-J, MoCA-J, H-Y stage, MDS-UPDRS parts I-V, PDQ-8, and biochemical factors (TP, Alb) between the non-sarcopenia and sarcopenia groups. The chi-squared test was used to compare sex, the presence or absence of swallowing disturbance, the presence or absence of falls, and anemia between the non-sarcopenia and sarcopenia groups. Factors related to the non-sarcopenia and sarcopenia groups (age, sex, disease duration, MoCA-J, presence or absence of swallowing disturbance, H-Y stage, MDS-UPDRS parts I-IV total score, and PDQ-8 total score) were analyzed using logistic regression analysis. Statistical significance was set at *p* < 0.05.

## 3 Results

### 3.1 Patient background

We included 97 patients with PD (55 males and 42 females), with a mean age of 69.8 ± 9.6 years (44–89 years) and disease duration of 7.3 ± 6.0 years (0.5–26 years). The mean LEDD was 477.0 ± 306.2 mg/day, the mean MMSE-J was 28.5 points, the mean MoCA-J was 25.8 points (25 points or less: 36 patients), and the mean SDQ-J total score was 4.8 ± 4.7 (swallowing disturbance group: 13 patients). Furthermore, 12, 66, and 19 patients had H-Y stage I, II, and III, respectively. The mean MDS-UPDRS parts I–IV total scores were 10.2 ± 5.6, 11.3 ± 7.1, 15.0 ± 7.9, and 1.4 ± 2.1 for parts I, II, III, and IV, respectively. The mean PDQ-8 total score was 5.4 ± 4.4 (Table 1).

**TABLE 1:** Clinical characteristics of total patients with Parkinson’s disease.

Number of patients	97
Gender (male/female)	55/42
Age (years)	69.8 ± 9.6 (44–89)
Disease duration (years)	7.3 ± 6.0 (0.5–26)
LEDD	477.0 ± 306.2 (0–1670.4)
MMSE-J	28.5 ± 1.8 (24–30)
MoCA-J	25.8 ± 3.1 (16–30) 25 points or less: 36 patients
SDQ-J total score	4.8 ± 4.7 (0.5–23.5) swallowing disturbance group: 13 patients
Hoehn and Yahr stage	I: 12, II: 66, III: 19
MDS UPDRS part I total score	10.2 ± 5.6 (2–26)
Part II total score	11.3 ± 7.1 (0–32)
Part III total score	15.0 ± 7.9 (2–44)
Part IV total score	1.4 ± 2.1 (0–9)
PDQ-8 total score	5.4 ± 4.4 (0–22)

Values are mean ± SD (range). LEDD, levodopa equivalent daily dose; MMSE-J, Japanese version of Mini-Mental State Examination; MoCA-J, Japanese version of Montreal Cognitive Assessment; SDQ-J, Japanese version of Swallowing Disturbance Questionnaire; MDS-UPDRS, Movement Disorder Society Revision of the Unified Parkinson’s disease rating scale; PDQ-8, Parkinson’s Disease Questionnaire.

### 3.2 Patient backgrounds and clinical characteristics of the non-sarcopenia and sarcopenia groups

Patient backgrounds and clinical characteristics are listed in Table 2. The prevalence of sarcopenia was 33%, with 65 patients in the non-sarcopenia group (43 males and 22 females) and 32 patients in the sarcopenia group (12 males and 20 females). Patients with PD in the non-sarcopenia group had a mean age of 67.3 ± 10.1 years (44–89 years), disease duration of 7.4 ± 6.0 years (0.5–22 years), and H-Y stage of 7 patients I, 51 patients II, and 7 patients III. Patients with PD in the sarcopenia group had an age of 74.8 ± 5.7 years (60–86 years), disease duration of 7.1 ± 5.9 years (0.5–26 years), and H-Y stage of 5 patients I, 15 patients II, and 12 patients III. There were significant differences between the non-sarcopenia and sarcopenia groups in age and sex (female) (*p* < 0.05).

**TABLE 2:** Background and clinical characteristics of patients with Parkinson’s disease in the non-sarcopenia and sarcopenia groups.

	Non-sarcopenia group (*n* = 65, 67%)	Sarcopenia group (*n* = 32, 33%) (Severe sarcopenia group: *n* = 18)	*p*-value
Gender (male/female)	43/22	12/20	0.007*
Age (years)	67.3 ± 10.1 (44–89)	74.8 ± 5.7 (60–86)	0.0002
Disease duration (years)	7.4 ± 6.0 (0.5–22)	7.1 ± 5.9 (0.5–26)	0.83
LEDD	496.5 ± 321.8 (0–1670.4)	438.6 ± 272.5 (100–1420)	0.32
SDQ-J total score	3.8 ± 3.5 swallowing disturbance group: five	6.8 ± 6.0 swallowing disturbance group: eight	0.019*
Groups with and without falls	without falls: 42, with falls: 23	without falls: 19, with falls: 13	0.62
Hoehn and Yahr stage	I: 7, II: 51, III: 7	I: 5, II: 15, III: 12	0.06
MDS-UPDRS part I	9.7 ± 5.2	11.4 ± 6.2	0.18
MDS-UPDRS part II	10.5 ± 6.5	13.0 ± 8.1	0.19
MDS-UPDRS part III	13.7 ± 6.4	17.8 ± 10.0	0.027
Arising from chair	0.4 ± 0.6	0.8 ± 0.8	0.049
Postural stability	0.8 ± 0.9	1.3 ± 1.0	0.006
Global spontaneity of movement	1.0 ± 0.6	1.3 ± 0.6	0.013
MDS-UPDRS part IV Groups with and without dyskinesia	1.5 ± 2.3 without dyskinesia: 52, with dyskinesia: 13	1.1 ± 1.9 without dyskinesia: 29, with dyskinesia: 3	0.48
PDQ-8 total score	5.2 ± 4.5	5.7 ± 4.3	0.43

Values are mean ± SD (range), Wilcoxon test, *Chi-square test. LEDD, levodopa equivalent daily dose; SDQ-J, Japanese version of Swallowing Disturbance Questionnaire; MDS-UPDRS, Movement Disorder Society Revision of the Unified Parkinson’s disease rating scale; PDQ-8, Parkinson’s Disease Questionnaire.

### 3.3 Comparison of SDQ, fallings, MDS-UPDRS, PDQ-8, blood test between non-sarcopenia and sarcopenia group

The results in Table 2 show a significant difference between the non-sarcopenia and sarcopenia groups in the SDQ-J total score. A comparison of falls and the presence of sarcopenia showed no significant difference in falls between the two groups.

**FIGURE 1:**
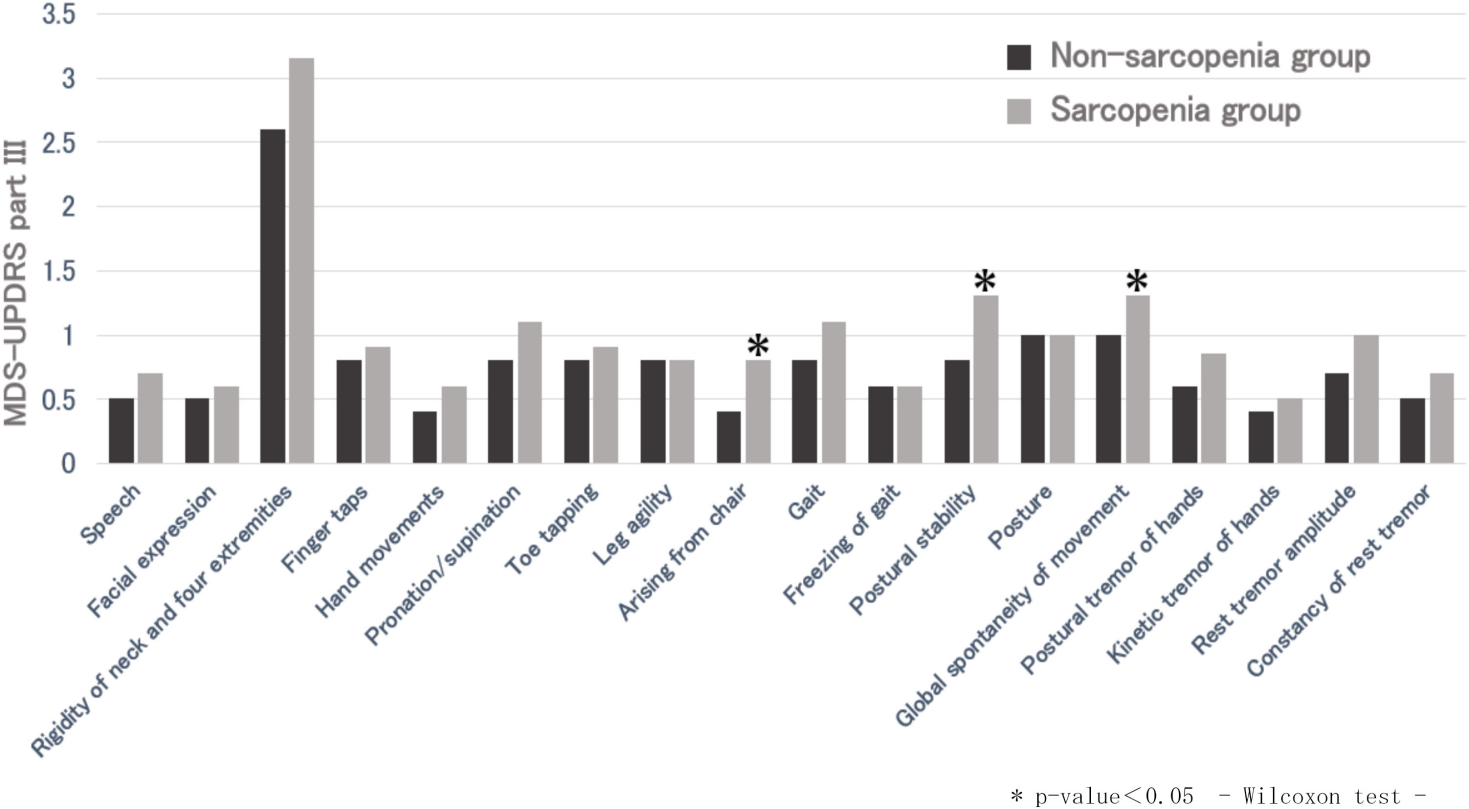
Comparison of MDS-UPDRS part III between non-sarcopenic and sarcopenic groups. *p-value < 0.05, Wilcoxon test.

The MDS-UPDRS part III total score was significantly higher in the sarcopenia group than the non-sarcopenia group. The mean part III total score was 13.7 ± 6.4 in the non-sarcopenia group and 17.8 ± 10.0 in the sarcopenia group. There were significant differences in the part III sub-items, “arising from a chair,” “postural stability,” and “global spontaneity of movement.” In addition, among the sarcopenia assessment items (grip strength, five-time chair stand test, and skeletal muscle mass), a significant difference was observed between the part III total score and grip strength (*p* = 0.0051) (Figure 1). Grip strength also showed significant differences in several part III subitems, including hand movements, toe tapping, arising from a chair, postural stability, global spontaneity of movement, postural tremor, and kinetic tremor. Furthermore, there was no significant difference in the MDS-UPDRS part I, II, and IV total scores between the two groups; however, the sub-items of part I “cognitive impairment” and the part II sub-item “personal hygiene” were significantly different (*p* < 0.05).

There was no significant difference between the non-sarcopenia and sarcopenia groups in the PDQ-8 total score and the PDQ-8 sub-items. There was no significant difference between the non-sarcopenia and sarcopenia groups in TP (*p* = 0.98), Alb (*p* = 0.85), and Anemia (*p* = 0.70) by blood test.

### 3.4 Differences in cognitive function between non-sarcopenic and sarcopenic groups

In the comparison of cognitive function between the non-sarcopenia and sarcopenia groups, a significant difference was observed in the cognitive impairment of the MDS-UPDRS part I sub-items (Table 3). In contrast, there were no significant differences between the two groups in the MMSE-J or MoCA-J scores (Table 3).

**TABLE 3:** Differences in cognitive function between non-sarcopenic and sarcopenic groups.

	Non-sarcopenia group	Sarcopenia group	*p*-value
MMSE	28.8 ± 1.3 (26–30)	27.7 ± 2.5 (24–30)	0.07
MoCA-J	26.5 ± 2.0 (21–30) 25 points or less: 21 patients (32.3%)	24.4 ± 4.3 (16–30) 25 points or less: 15 patients (46.9%)	0.08
MDS-UPDRS part I sub-item: cognitive impairment	0.4 ± 0.5	0.9 ± 1.0	0.02

Values are mean ± SD (range). Wilcoxon test. MMSE-J, Japanese version of Mini-Mental State Examination; MoCA-J, Japanese version of Montreal Cognitive Assessment; MDS-UPDRS, Movement Disorder Society Revision of the Unified Parkinson’s disease rating scale.

### 3.5 Factors associated with sarcopenia

Factors contributing to sarcopenia were examined, including patient background (age, sex, disease duration, MoCA-J, SDQ-J total score), H-Y stage, MDS-UPDRS parts I–IV total scores, and PDQ-8 total score were examined, and the presence of sarcopenia was associated with gender (female), cognitive impairment, and swallowing disturbance (Table 4).

**TABLE 4:** Factors associated with sarcopenia.

	Estimated value	Standard error of the mean	*t*-value	*p*-value
Age (years)	0.08	0.04	3.28	0.07
Sex (1. male /2. female)	−0.73	0.29	6.51	0.011
Disease duration (years)	−0.04	0.06	0.48	0.49
MoCA-J	−0.24	0.10	5.65	0.017
SDQ-J total score	0.19	0.08	4.67	0.031
Hoehn and Yahr stage	−0.32	0.58	0.31	0.58
MDS-UPDRS part I total score	−0.03	0.08	0.11	0.74
Part II total score	0.03	0.06	0.19	0.66
Part III total score	0.06	0.05	1.86	0.17
Part IV total score	−0.03	0.16	0.05	0.83
PDQ-8 total score	−0.09	0.11	0.67	0.41

Method of least square. MoCA-J, Japanese version of Montreal Cognitive Assessment; SDQ-J, Japanese version of Swallowing Disturbance Questionnaire; MDS-UPDRS, Movement Disorder Society Revision of the Unified Parkinson’s disease rating scale; PDQ-8, Parkinson’s Disease Questionnaire.

## 4 Discussion

The general prevalence of sarcopenia is reportedly 7.5%–13.8%, depending on the definition and data used (Akune et al., 2014; Yoshida et al., 2014; Yoshimura et al., 2017). In this study, the prevalence of sarcopenia in patients with PD was 33.0%, indicating a significantly higher prevalence than that in healthy persons. In this study, it was difficult to classify sarcopenia as primary or secondary; however, disuse muscle atrophy and weakness, which occur as secondary disorders in patients with PD, could be considered secondary sarcopenia due to “reduced activity,” according to the European Working Group on Sarcopenia in Older People (EWGSOP) (Cruz-Jentoft et al., 2010). In addition, some participants may have shown decreased performance on the five-time chair stand test and reduced grip strength due to insufficient dopaminergic treatment. In general, adequate dopaminergic therapy is defined as LEDD of approximately 600–800 mg/day or higher, according to a previous report (Tomlinson et al., 2010). In this study, the mean LEDD was 476.6 ± 306.4 mg/day, which may indicate that some patients did not receive an adequate dopaminergic dose.

In the comparison of the non-sarcopenic and sarcopenic group, MDS-UPDRS part I, which assesses subjective non-motor symptoms, and part II, which assesses subjective motor symptoms, were not significantly different between the two groups, suggesting that the presence of sarcopenia and subjective symptoms in patients with PD are poorly related. In this study, patients with sarcopenia showed higher the part III total scores than those without sarcopenia, particularly in subitems related to lower-limb function such as “arising from a chair” and “postural stability.” In addition, grip strength showed significant differences with the part III total score and several subitems, suggesting that overall motor severity is closely linked to reduced muscle strength. Based on these findings, sarcopenia in patients with PD is primarily associated with motor impairment rather than non-motor symptoms, underscoring the importance of assessing and maintaining muscle strength in clinical management. Part IV was not significantly different between the non-sarcopenia and sarcopenia group, suggesting that sarcopenia is not associated with motor complications.

Both the PDQ-8 total score and sub-items were not significantly different between the non-sarcopenic and sarcopenic groups of patients with PD in this study, suggesting that the presence of sarcopenia is poorly related to QoL in patients with PD. The EWGSOP defines sarcopenia as “a syndrome characterized by a progressive and generalized loss of muscle mass and strength, with risk of physical dysfunction, reduced QoL, and death” (Cruz-Jentoft et al., 2010). Although sarcopenia is associated with QoL in older Japanese patients (Tanimoto et al., 2012), there have been no previous reports on sarcopenia and QoL in patients with PD. Previous studies examining the PDQ-8 and MDS-UPDRS found that the PDQ-8 was correlated with parts I and II of the MDS-UPDRS, suggesting an association between QoL and subjective symptoms (Skorvanek et al., 2018). It was presumed that subjective motor and non-motor symptoms of PD may be more related to QoL in patients with PD than to the loss of muscle mass or strength.

The comparison of cognitive impairment between the non-sarcopenia and sarcopenia groups showed significant differences in MDS-UPDRS part I cognitive impairment. Cognitive impairment is one of the common non-motor symptoms in patients with PD and can affect attention, executive function, and visuospatial abilities (Aarsland et al., 2010). These cognitive deficits may lead to reduced physical activity and poor nutritional intake, thereby contributing to the development and progression of sarcopenia. An association between sarcopenia and cognitive impairment has been reported (Cabett Cipolli et al., 2019), and sarcopenia has been associated with mild cognitive impairment and depression in older women (Lee et al., 2018), and some reports have shown the effect of malnutrition on cognitive impairment in older adults (Petersson and Philippou, 2016). Sequential lifestyle changes, including impaired exercise and inactivity, may be associated with sarcopenia and cognitive impairment (Cabett Cipolli et al., 2019), a finding that also supports the previous reports of sarcopenia in patients with PD in this study.

In this study, sarcopenia in patients with PD was most strongly associated with being female, cognitive impairment, and swallowing disturbance. Some studies have reported no sex differences in sarcopenia in patients with PD (Cai et al., 2021; Ponsoni et al., 2023), whereas others have reported that sex differences in women are independently associated with sarcopenia (Lima et al., 2020). Androgens are important in the maintenance of muscle mass, and low plasma testosterone levels may cause or accelerate muscle- and age-related diseases, such as sarcopenia (Basualto-Alarcón et al., 2014; Wu et al., 2020). The association between sex, female hormones and PD has long been indicated; (Kumagai et al., 2014; Cerri et al., 2019) however, the relationship between the presence or absence of sarcopenia and sex in patients with PD is controversial and has not been established in the literature. These results also suggest that cognitive impairment and swallowing disturbances are associated with sarcopenia in patients with PD, even in the absence of undernutrition. In older adults, an association between swallowing and chewing functions has been reported, along with generalized sarcopenia (Shiozu et al., 2015; Murakami et al., 2015). The mechanism underlying swallowing disturbance caused by sarcopenia is considered to be secondary sarcopenia of the whole body and swallowing-related muscles due to inactivity, malnutrition, and disease (Wakabayashi and Sakuma, 2014). Although data on the association between sarcopenia and swallowing disturbance in patients with PD are lacking, our results suggest a link between the swallowing disturbances caused by the disease and sarcopenia. The results of this study also suggest the importance of assessing muscle strength and evaluating sarcopenia, particularly in patients with PD who have being female, cognitive impairment, or swallowing disturbance.

This study has some limitations. The presence of sarcopenia prior to the diagnosis of PD could not be evaluated, and the medical background of the patients with PD could not be considered; therefore, it is undeniable that there may have been a bias in the cases. Moreover, although an association between PD severity and sarcopenia has been reported previously (Vetrano et al., 2018), this study did not include H-Y stage IV and thus could not evaluate patients with severe PD. In addition, the motor score (MDS-UPDRS part III) was evaluated in the “on” state and was not compared with that in the “off” state, warranting consideration in future studies.

## 5 Conclusion

The prevalence of sarcopenia in patients with mild to moderate PD was higher than in the general population, and the most contributing factors were being female, cognitive impairment, and swallowing disturbance. In clinical management, it is important to assess muscle strength and evaluate sarcopenia, particularly in patients with such risk factors.

## Data Availability

The datasets presented in this study can be found in online repositories. The names of the repository/repositories and accession number(s) can be found in the article/Supplementary material.
