# Supplementary figures and images for: Clinical analysis of sarcopenia prevalence and its influencing factors in patients with Parkinson’s disease
Source: Front Aging Neurosci. 2025 Dec 8;17:1718723. doi: 10.3389/fnagi.2025.1718723 (PMC12719491; doi:10.3389/fnagi.2025.1718723)

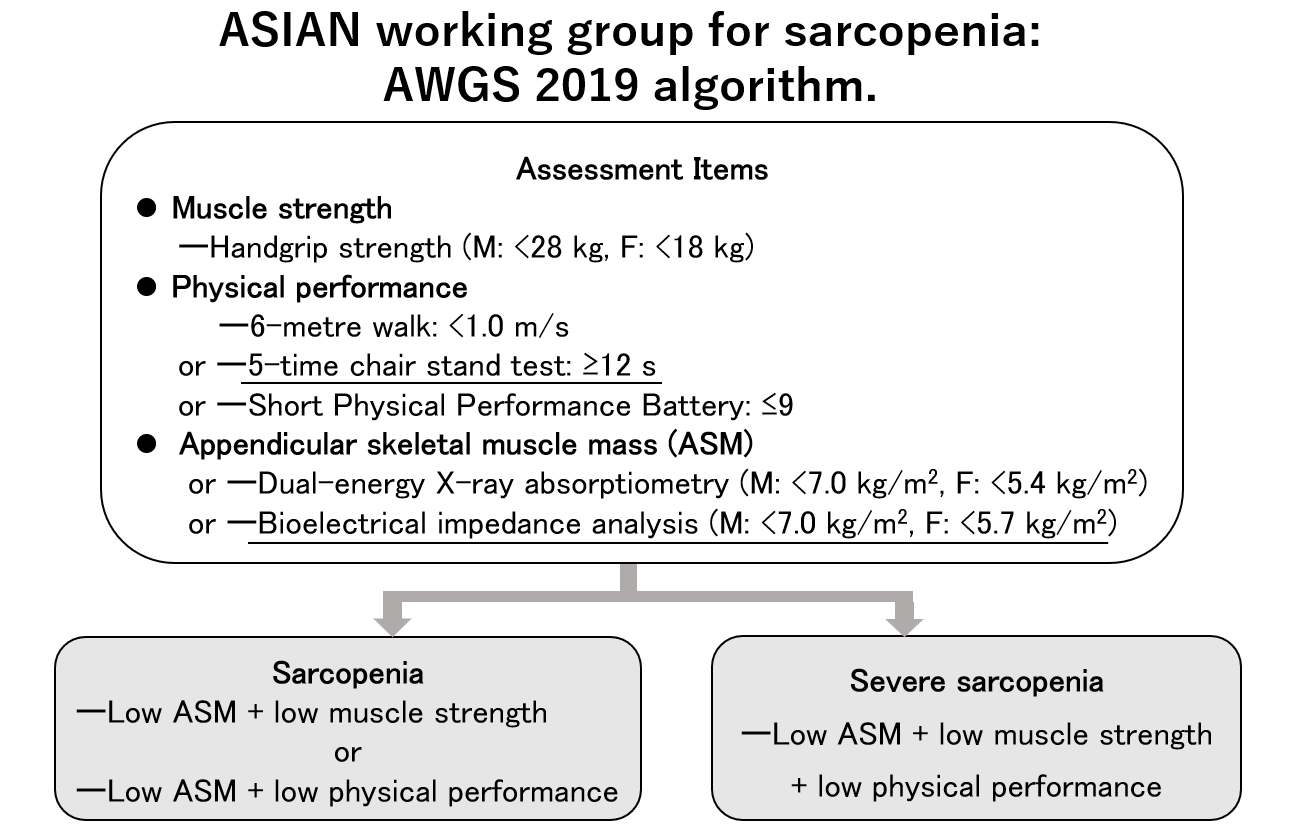
 Figure 1. ASIAN working group for sarcopenia: AWGS 2019 algorithm. M, male; F, female.

Supplement: Supplementary file 1 [file Data_Sheet_1.docx]
