# Supplementary material for: Clinical analysis of sarcopenia prevalence and its influencing factors in patients with Parkinson’s disease
Source: Front Aging Neurosci. 2025 Dec 8;17:1718723. doi: 10.3389/fnagi.2025.1718723 (PMC12719491; doi:10.3389/fnagi.2025.1718723)
Supplement: Supplementary file 2 [file Data_Sheet_2.docx]

| Swallowing disturbance questionnaire (SDQ)  Question: | 0 | 1 | 2 | 3 |
| --- | --- | --- | --- | --- |
|  | **Never** | **Seldom**  **(once a month or less)** | **Frequently**  **(1-7 times a week)** | **Very frequently (more than 7 times a week)** |
| Q1. Do you experience difficulty chewing solid food like an apple, cookie, or a cracker? |  |  |  |  |
| Q2. Are there any food residues in your mouth, in your cheeks, under your tongue or stuck to your palate after swallowing? |  |  |  |  |
| Q3. Does food or liquid come out of your nose when you eat or drink? |  |  |  |  |
| Q4. Does chewed-up food dribble from your mouth? |  |  |  |  |
| Q5. Do you feel you have too much saliva in your mouth; do you drool or have difficulty swallowing your saliva? |  |  |  |  |
| Q6. Do you swallow chewed-up food several times before it goes down your throat? |  |  |  |  |
| Q7. Do you experience difficulty in swallowing solid food (i.e., do apples or crackers get stuck in your throat)? |  |  |  |  |
| Q8. Do you experience difficulty in swallowing pureed food? |  |  |  |  |
| Q9. While eating, do you feel as if a lump of food is stuck in your throat? |  |  |  |  |
| Q10. Do you cough while swallowing liquids? |  |  |  |  |
| Q11. Do you cough while swallowing solid foods? |  |  |  |  |
| Q12. Immediately after eating or drinking, do you experience a change in your voice, such as hoarseness or reduced? |  |  |  |  |
| Q13. Other than during meals, do you experience coughing or difficulty breathing as a result of saliva entering your windpipe? |  |  |  |  |
| Q14. Do you experience difficulty in breathing during meals? |  |  |  |  |
| Q15. Have you suffered from a respiratory infection (pneumonia, bronchitis) during the past year? | **No 0.5** | **Yes 2.5** |  |  |

Figure 2. Swallowing Disturbance Questionnaire (SDQ)
