# Supplementary material for: Clinical analysis of sarcopenia prevalence and its influencing factors in patients with Parkinson’s disease
Source: Front Aging Neurosci. 2025 Dec 8;17:1718723. doi: 10.3389/fnagi.2025.1718723 (PMC12719491; doi:10.3389/fnagi.2025.1718723)
Supplement: Supplementary file 3 [file Data_Sheet_3.docx]

| Parkinson's Disease Questionnaire (PDQ-8)：  Due to having Parkinson’s disease, how often during the last month have you... |
| --- |
| Q1 Had difficulty getting around in public?  　Q2 Had difficulty dressing yourself?  　Q3 Felt depressed?  　Q4 Had problems with your close personal relationships?  　Q5 Had problems with your concentration, e.g. when reading or watching TV?  　Q6 Felt unable to communicate with people properly?  　Q7 Had painful muscle cramps or spasms?  　Q8 Felt embarrassed in public due to having Parkinson’s disease? |

Figure 3. Parkinson's disease questionnaire (PDQ-8)
